# Leaving no one behind: a neglected tropical disease indicator and tracers for the Sustainable Development Goals

**DOI:** 10.1093/inthealth/ihw002

**Published:** 2016-03-03

**Authors:** Christopher Fitzpatrick, Dirk Engels

**Affiliations:** Department of Control of Neglected Tropical Diseases, World Health Organization, Geneva, Switzerland

**Keywords:** Control, Elimination, Eradication, Monitoring, Neglected tropical diseases, Sustainable Development Goals

## Abstract

The Sustainable Development Goals (SDGs) have emerged as a global pledge to ‘leave no one behind’. Under SDG 3, ‘Ensure healthy lives and promote wellbeing for all’, target 3.3 extends the Millennium Development Goals (MDGs) beyond HIV, TB and malaria to ‘end the epidemic’ of neglected tropical diseases (NTDs) by 2030. Other targets are also relevant to NTDs, especially 3.8 (Universal Health Coverage), 6.1 (water) and 6.2 (sanitation). This commentary summarises the proposed NTD indicator (3.3) and tracers (3.8 and 6.1/6.2). These will help ensure that the world's poorest and most marginalized people are prioritized at every step on the path towards SDG targets.

## Overview of neglected tropical diseases in the Sustainable Development Goals

In September 2015, the United Nations formally endorsed a sustainable development agenda.^1^ The 17 Sustainable Development Goals (SDGs) have emerged as a global pledge to ‘leave no one behind’. Meanwhile, the neglected tropical diseases (NTDs) have long been defined by their burden on the poorest and most marginalized populations. SDG 3, ‘Ensure healthy lives and promote wellbeing for all at all ages’, has nine targets, one of which extends the Millennium Development Goals (MDGs) beyond HIV, TB and malaria to ‘end the epidemic’ of NTDs by 2030 (target 3.3).

The target (3.8) for Universal Health Coverage (UHC) is very relevant to NTDs, calling for ‘access to quality essential health-care services and access to safe, effective, quality and affordable essential medicines and vaccines for all.’ NTD medicines on WHO's Model Lists of Essential Medicines^2^ have been donated by the pharmaceutical industry and should be prioritized for delivery under UHC. Access to water and sanitation for all, with dedicated targets 6.1 and 6.2, respectively, is also recognized as critical to accelerating and sustaining progress on NTDs.^3^

As an observer of the Inter-Agency and Expert Group on SDG indicators (IAEG-SDGs), WHO was invited to provide input to the process of indicator development led by the United Nations Statistical Commission and Member States. This commentary summarises WHO's proposal for a global NTD indicator for target 3.3 and NTD tracers of equity for targets 3.8 and 6.1/6.2. The tracers are conceived as a contribution of the NTD community to monitoring equity in progress towards other development goals and targets, including those of other sectors.

## The NTD indicator for SDG target 3.3

WHO's proposed NTD indicator is a synthesis of existing NTD targets with political endorsement in the most recent World Health Assembly resolution on NTDs, adopted in 2013.^4^ The resolution refers to targets set out in WHO's roadmap for accelerating work to overcome the global impact of NTDs.^5^ The roadmap includes targets to eradicate or eliminate 11 diseases, globally or regionally, by 2020. It outlines the five main interventions required to achieve those targets: preventive chemotherapy (PC); case detection and management; vector control; veterinary public health; and water, sanitation and hygiene. Health systems strengthening, especially capacity-building of volunteers, community leaders and front-line health workers, is recognized to cut across those interventions.

Progress towards NTD roadmap targets is already being measured and reported to WHO as disease-specific indicators. The objective for SDG target 3.3 was to maintain (at least) one global NTD indicator under a target already crowded with indicators for HIV, TB, malaria and hepatitis. The challenge was to achieve consensus on a single indicator for ‘the end’ of NTDs as a group, without undermining existing disease-specific indicators. After consultation with international NTD experts and partners from civil society^6^ consensus emerged around the indicator ‘Number of people requiring interventions against NTDs’. After further technical consultation, the details (metadata) of that indicator are now available in Box 1.

**Box 1.** Indicator metadata submitted to the Inter-Agency and Expert Group on Sustainable Development Goal indicators

**Target:**	3.3: By 2030, end the epidemics of AIDS, TB, malaria and neglected tropical diseases (NTDs) and combat hepatitis, water-borne diseases and other communicable diseases.
**Abbreviated indicator name:**	Number of people requiring interventions against neglected tropical diseases.
**Indicator name:**	Number of people requiring treatment as a tracer for interventions against neglected NTDs.
**Domain:**	Health status.
**Subdomain:**	Infectious disease.
**Associated terms:**	Neglected tropical diseases; eradication; elimination; control.
**Definition:**	Number of people requiring treatment as a tracer for interventions against any one of the NTDs targeted by WHO, including 11 NTDs targeted for eradication or elimination in World Health Assembly resolutions. Treatment is broadly defined to allow for preventive, curative, surgical or rehabilitative treatment. Other (non-treatment) interventions (e.g. surveillance, disability and inclusion) are to be addressed in the context of targets and indicators for Universal Health Coverage (UHC).
**Numerator:**	Average annual number of people requiring preventive chemotherapy (PC) for at least one PC-NTD; plus population at risk requiring mass treatment for yaws eradication; plus 3. number of new cases requiring individual treatment (disease management) for other NTDs.
**Denominator:**	Not applicable.
**Disaggregation/additional dimension:**	Disaggregation by intervention and disease is required; ending the epidemic of NTDs requires a reduction in the number of people requiring interventions for each NTD. Disaggregation by age is required for PC: pre-school-aged children (1–4 years), school-aged (5–14 years) and adults (≥15 years). Based on current reporting systems, disaggregation by sex and urban/rural is optional or depends on which diseases are co-endemic.
**Method of measurement:**	The number of people requiring treatment against NTDs is measured by existing country systems, and reported through joint request and reporting forms (http://www.who.int/neglected_diseases/preventive_chemotherapy/reporting/en/) for donated medicines, and the integrated NTD database (http://www.who.int/neglected_diseases/data/ntddatabase/en/).
**Method of estimation:**	Some estimation is required to aggregate data across interventions and diseases. Average annual number of people requiring PC for at least one PC-NTD: people may require PC for more than one PC-NTD. The number of people requiring PC is compared across the PC-NTDs, by age group and implementation unit (e.g. district). The largest number of people requiring PC is retained for each age group in each implementation unit. The total is considered to be a conservative estimate of the number of people requiring PC for at least one PC-NTD. Prevalence surveys (e.g. transmission assessment surveys; http://www.who.int/lymphatic_filariasis/resources/TAS_training_materials/en/) determine when an NTD has been eliminated or controlled and PC can be stopped or reduced in frequency, such that the average annual number of people requiring PC is reduced. Population at risk requiring mass treatment for yaws eradication: populations at risk have been estimated in countries known to be endemic for yaws (see ‘Other possible data sources’ below); prevalence surveys will determine when a disease has been eliminated at the country level and the population is certified as yaws-free. Number of new cases requiring individual treatment for other NTDs: the number of new cases is based on country reports of new and known cases of Buruli ulcer, Chagas disease, cysticercosis, dengue, echinococcosis, human African trypanosomiasis (HAT), leprosy, the leishmaniases and rabies. Where the number of people requiring and requesting surgery for PC-NTDs (e.g. trichiasis or hydrocele surgery) is reported, it can be added here. Similarly, new cases requiring and requesting rehabilitation (e.g. leprosy or lymphoedema) could be added. Case reports may not be comparable over time; estimation may be required to adjust for changes in case-finding and reporting. Note 1: populations referred to under 1, 2 and 3 may overlap; the sum would overestimate the total number of people requiring treatment. The maximum of 1, 2 and 3 is retained at the lowest common implementation unit and summed to get conservative country, regional and global aggregates. By 2030, improved co-endemicity data and models will validate the trends obtained using this simplified approach. Note 2: the target for eradication of dracunculiasis remains 2015; it is not included in this indicator for the 2030 target.
**Measurement frequency:**	Annual.
**Monitoring and evaluation framework:**	Impact.
**Preferred data sources:**	Country data are published via the Global Health Observatory (http://www.who.int/gho/neglected_diseases/en/) and PC databank (http://www.who.int/neglected_diseases/preventive_chemotherapy/databank/en/).
**Other possible data sources:**	Atlas of human African trypanosomiasis (http://www.who.int/trypanosomiasis_african/country/risk_AFRO/en/). Weekly epidemiological record (Chagas disease: http://www.who.int/wer/2015/wer9006.pdf). Regional databases (dengue: http://www.paho.org/hq/index.php?option=com_topics&view=rdmore&cid=6290&Itemid=40734&lang=en). Systematic reviews (yaws: http://dx.doi.org/10.1016/S2214-109X(15)00011-X).
**Further information and related links:**	WHO. Global plan to combat neglected tropical diseases, 2008–2015. Geneva: World Health Organization; 2007. http://whqlibdoc.who.int/hq/2007/who_cds_ntd_2007.3_eng.pdf [accessed 29 March 2015]. WHO. Accelerating work to overcome the global impact of neglected tropical diseases. Geneva: World Health Organization; 2012. http://www.who.int/neglected_diseases/NTD_RoadMap_2012_Fullversion.pdf [accessed 29 March 2015]. WHO. Investing to overcome the global impact of neglected tropical diseases. Geneva: World Health Organization; 2015. http://www.who.int/neglected_diseases/9789241564861/en/ [accessed 29 March 2015].

The feasibility of NTD elimination and control has been demonstrated in individual countries. For example, years of high-quality intervention resulted, by 2014, in the interruption of transmission and an end to mass treatment of lymphatic filariasis in 18 countries.^7^ In the third WHO NTD report, it was projected that if NTD elimination and control targets are met globally, the average number of people requiring PC would decrease by about 90% globally between 2015 and 2030.^8^ It is not quite 100% because treatment for some diseases is projected to be reduced in frequency, not stopped.

At least 149 countries are thought to require interventions against NTDs, including PC as well as curative treatment or disease management. The metadata allow for a broad definition of treatment, including also surgical and rehabilitative treatment, where quality data are available. Given that PC seeks to treat entire communities rather than individual cases, the global indicator will be dominated (numerically) by requirements for PC. Disaggregation by intervention (preventive, curative, etc.) and disease is, therefore, needed to monitor progress towards the end of (all) NTDs and, ultimately, tell a more nuanced story of successes and failures, as required.

Non-treatment interventions (e.g. surveillance, disability management and efforts to foster inclusion), including some which will be required well beyond 2020, are better addressed in the context of target 3.8, which we turn to now.

## The NTD tracers of equity for SDG targets 3.8 and 6.1/6.2

In WHO's proposed indicator for SDG target 3.8, NTD PC is listed alongside antiretroviral therapy, TB treatment and insecticide-treated bed net use as a tracer intervention for UHC (there is also an indicator for financial protection [‘fraction of the population protected against catastrophic/impoverishing out-of-pocket health expenditure’] but this will be measured across all diseases and no tracer interventions have been proposed). However, it could play a special role in monitoring the equity or fairness of UHC reform. If NTD PC coverage remains low, then UHC reform may be failing to prioritize cost-effective interventions for the poorest and most marginalized populations. The first WHO/World Bank report on monitoring UHC states that ‘Monitoring preventive chemotherapy coverage remains key to ensuring that the diseases of the least well-off are being prioritized from the very beginning of the path towards UHC.’^9^

That report focussed on PC coverage due to the availability of data from many countries. Data on coverage with other NTD interventions exists but is more region- or country-specific. The NTD tracer could be broadened to monitor the coverage with a basic package of NTD interventions—for example, the proportion of the population living in districts implementing a basic package of interventions against locally endemic NTDs. The basic package of interventions—essential interventions for which coverage can be measured—remains to be defined. But target 3.8 would be the best place to deal with longer-term and more systemic interventions, including those dealing with surveillance and disability and inclusion.

Water, sanitation and hygiene (WASH) is another longer-term and more systemic intervention. Access to water and sanitation for all (SDG 6) is to be tracked by the percentage of the population using safely managed drinking water services (under target 6.1) and safely managed sanitation services (under target 6.2). Though these indicators reside outside of the ‘health’ goal (SDG 3), progress towards them will decrease the number of people living in conditions favorable to the transmission of NTDs and requiring treatment against them (under target 3.3).

WHO's global NTD and WASH strategy 2015–2020 for ‘accelerating and sustaining progress on NTDs’ includes among its strategic objectives ‘to use WASH and NTD monitoring to highlight inequalities, target investment, and track progress’. Joint monitoring ‘helps the WASH sector achieve its goal of universal access by targeting investments to the poorest and most marginalized populations.’^3^ A methodological note prepared by WHO and UNICEF for SDG 6 targets and indicators encourages subnational monitoring of WASH coverage in particularly vulnerable areas, such as NTD-endemic districts.^10^

## Beyond SDGs 3 and 6

By the time this commentary is published, the IAEG-SDGs will have already submitted its proposal to the 47th session of the United Nations Statistical Commission on 30 November 2015. A global indicator framework for the SDGs will be agreed by the UNSC by March 2016 and adopted thereafter by the UN Economic and Social Council (ECOSOC) and General Assembly. If included, the NTD indicator and tracers will help ensure that the world's poorest and most marginalized populations are prioritized at every step on the path toward SDGs 3 and 6.

The NTD community now has to operationalize its proposed indicator, deciding what ‘the end’ of NTDs by 2030 means in terms of the number of people requiring interventions—globally as well as for individual countries and NTDs. Based on the projections mentioned above, a ‘90% reduction in the number of people requiring interventions’ could be one milestone for success at the global level. 100% coverage with a basic package of NTD interventions and 100% WASH coverage in NTD endemic districts would be logical extensions of targets 3.8 and 6.1/6.2.

Of course, health is also closely linked to many other SDGs, including ending poverty (SDG 1), ending hunger (SDG 2), ensuring quality education (SDG 4) and combating climate change and its impacts (SDG 13). Achievement of those goals will certainly facilitate progress towards the NTD target. NTD tracers of equity could also help target investments toward those goals, for maximum return. Where there are NTDs, there is more poverty, hunger, school absenteeism and vulnerability to the environment.

SDG 17 on global partnership connects with all other goals through targets related to finance (the first being domestic finance) and data, among others. In the NTD space, innovative mechanisms are being explored that finance outputs and outcomes rather than inputs, but these depend on high-quality, timely and reliable data. NTD data should be disaggregated, whenever possible, by income, gender, age, disability, geographic location and other characteristics.

We do not share the opinion expressed elsewhere that the main outcome of the inclusion of NTDs within the SDGs will be more money for NTDs.^6^ The proposal of a single global NTD indicator reflects our community's aspiration to deliver a package of interventions centered on the poorest and most marginalized. The proposal of NTD tracers for UHC and water and sanitation reflects our community's aspiration to advance the broader sustainable development agenda. An NTD indicator will not lead mechanically to more investment in interventions against NTDs. NTD tracers for UHC and water and sanitation could lead strategically to smarter investment of the money that will be made available for sustainable development over the next 15 years.
